# Guide Wire Induced Cardiac Tamponade: The Soft J Tip Is Not So Benign

**DOI:** 10.1155/2016/1436924

**Published:** 2016-08-11

**Authors:** Sankalp Dwivedi, Fayez Siddiqui, Milan Patel, Shaun Cardozo

**Affiliations:** ^1^St. Mary Mercy Livonia, Department of Internal Medicine, Livonia, MI 48154, USA; ^2^Wayne State University Detroit Medical Center, Detroit, MI 48201, USA

## Abstract

Central venous catheter (CVC) insertion rarely causes cardiac tamponade due to perforation. Although it is a rare complication, it can be lethal if not identified early. We report a case of cardiac tamponade caused by internal jugular (IJ) central venous catheter (CVC) insertion using a soft J-tipped guide wire which is considered safe and rarely implicated with cardiac tamponade. A bedside transthoracic echocardiogram (TTE) revealed a pericardial effusion with tamponade. An emergent bedside pericardiocentesis was done revealing bloody fluid and resulted in clinical stabilization.

## 1. Introduction

Literature advocates the use of J-tipped guide wires as they are considered to be safe due to their unique shape [1, 2]. They were introduced originally as they were associated with less likelihood of complications [1, 2]. Although rarely seen, one of the feared complications of CVC insertion is cardiac tamponade [1]. Due to the fatal nature of the illness, it should be suspected in any patient who deteriorates soon after the CVC insertion [3]. It is more common in neonates and pediatric age group with overall adult rates less than 1% [1, 4]. Although right atrium and right ventricle are commonly affected resulting in cardiac tamponade, it could also be due to superior vena cava perforation [3]. Carotid artery injury with retrograde dissection leading to cardiac tamponade as a complication of internal jugular vein catheterization has also been described in the literature [5]. The majority of the cases are due to catheter insertion; cardiac tamponade due to guide wire is extremely rare. There have been 5 cases of similar complications that were caused by Seldinger guide wire, at least 4 of those by J-tipped guide wire (Table 1) [2, 5, 6]. J-tipped guide wire is considered to be safe from the fatal complication of cardiac tamponade, but no data regarding its safety is currently available [1]. We report the first case of cardiac tamponade due to the J-tipped guide wire in a patient of thrombotic thrombocytopenic purpura (TTP).

## 2. Case

A 51-year-old female with past medical history of acute myelogenous leukemia status after bone marrow transplant in September 2013 was initially admitted for respiratory distress. She was started on broad spectrum antibiotics. Her hospital course was complicated by acute renal failure, uremia, and thrombocytopenia. After ADAMS13 had come back low, to initiate plasmapheresis for suspected TTP was decided. A Quinton catheter was decided to be placed using a soft J guide wire. Soon after the J-tipped guide wire was inserted, she became hypotensive, tachycardic, and nonresponsive. An urgent bedside echocardiogram showed a large circumferential pericardial effusion with right ventricular diastolic collapse, dilated and plethoric IVC and Doppler data compatible with a hemodynamically significant pericardial effusion. An echocardiogram a couple of days earlier had no evidence of pericardial fluid. The patient underwent pericardiocentesis, removing 600 cc of bloody fluid. Analysis of pericardial fluid was consistent with hemorrhagic pericardial tamponade with one nucleated cell and 320,000 red blood cells (RBCs).

## 3. Discussions

Due to the increased availability of CVC and ease of placement, it is one of the most frequent procedures to be performed in all age groups [7]. CVC insertion is associated with its complications during either the insertion or its maintenance. These include infection, pneumothorax, hemothorax, and thrombosis [7].

Extremely rare but possibly the most dreaded complication of CVC insertion is cardiac tamponade. Puncture of the cardiac wall seems to be the most widely recognized cause but infrequently superior vena cava (SVC) or even inferior vena cava (IVC) has additionally been implicated [1].

The J-tipped guide wire rarely causes perforation due to its J-tipped shape as it avoids the sharp tip of the guide wire coming in contact and perforating the structures on its way.

On the other hand, mechanism of pericardial effusion due to the catheter is poorly understood [1, 4]. In neonates, it has been hypothesized that it is likely due to myocardial weakness and areas, which are at increased risk of perforation [4]. For catheter-induced injury, movements of the catheter tip, angle of insertion, movement of the cardiac chambers, and direct trauma have all been postulated to be responsible for cardiac perforation [3].

In cases of injuries related to the guide wire, three of the previously reported cases had the end-stage renal disease (ESRD). In our case, the patient did not have prior ESRD but rather thrombotic thrombocytopenic purpura (TTP). To the best of our knowledge, this is the first case that has reported cardiac tamponade soon after the placement of the J-tipped guide wire in a patient with TTP. We believe both the renal failure and thrombocytopenia predisposed our patient to bleed easily; hence, even a small perforation by the guide wire has a higher chance of hemorrhagic complications leading to near fatal complication of cardiac tamponade [1].

Although the soft J-tipped guide wire is designed for decreased risk of perforation that is associated with the advancement of the wire, extreme caution must be taken in retraction of the wire especially in patients with bleeding diathesis due to the distal tip perforating the potential spaces at the vena cava-right atrial junctions and the right atrial free wall. Since the majority of the patients undergoing the procedure are critically ill, the diagnosis becomes difficult due to multiple comorbid conditions and sedation [3]. So, it is even more vital to recognize this complication as misdiagnosis may lead to worsening of the condition. Therefore, for any suspicion of such a complication, we endorse prompt evaluation with emergent ultrasound of the abdomen and heart to save valuable time. Proper strategies to avoid the risk of cardiac tamponade should be adopted which include performing the maneuver with fluoroscopy, avoiding inserting the guide wire, in most cases, for a length superior to 18 cm [8]. Also, avoidance to force the guide wire when met with resistance would be preventive against perforation of the cardiac chamber or the blood vessel depending upon the location of the tip. Although the J-tipped guide wires are usually considered safe, their safety may require reassessment specifically in cases of bleeding disorders.

## Figures and Tables

**Table 1:** 

Case	Site	Pathological evidence	Renal failure	Wire type	Bleeding diathesis	RV wall thickness	Site of perforation
1	Internal jugular	No	ESRD	Soft J	Yes	Normal	Right atrium
2	Internal jugular	No	ESRD	Soft J	Yes	Normal	Inferior vena cava
3	Subclavian	Yes	No	Soft J	No	Thin	Right ventricle
4	Internal jugular	No	ESRD	Soft J	Yes	Normal	Unknown
5	Internal jugular	No	No	Unknown	No	Normal	Carotid artery
